# Enhancing transparency and fairness in automated credit decisions: an explainable novel hybrid machine learning approach

**DOI:** 10.1038/s41598-024-75026-8

**Published:** 2024-10-24

**Authors:** Chioma Ngozi Nwafor, Obumneme Nwafor, Sanjukta Brahma

**Affiliations:** 1 Glasgow School for Business and Society, Department of Finance, Accountancy and Risk, Glasgow Caledonia University, Glasgow, Scotland; 2 School of Computing, Engineering and Built Environment, Glasgow Caledonian University, Glasgow, Scotland

**Keywords:** Credit risk, One-dimensional convolutional neural network, Shapley Additive explanation, Explainable artificial intelligence, And Logistics regression, Mathematics and computing, Computational science, Scientific data, Statistics

## Abstract

This paper uses a generalised stacking method to introduce a novel hybrid model that combines a one-dimensional convolutional neural network 1DCNN with extreme gradient boosting XGBoost. We compared the predictive accuracies of the proposed hybrid architecture with three conventional algorithms-1DCNN, XGBoost and logistic regression (LR) using a dataset of over twenty thousand peer-to-peer (P2P) consumer credit observations. By leveraging the SHAP algorithm, the research provides a detailed analysis of feature importance, contributing to the model’s predictions and offering insights into the overall and individual significance of different features. The findings demonstrate that the hybrid model outperforms the LR, XGBoost and 1DCNN models in terms of classification accuracy. Furthermore, the research addresses concern regarding fairness and bias by showing that removing potentially discriminatory features, such as age and gender, does not significantly impact the hybrid model’s classification capabilities. This suggests that fair and unbiased credit scoring models can achieve high effectiveness levels without compromising accuracy. This paper makes significant contributions to academic research and practical applications in credit risk management by presenting a hybrid model that offers superior classification accuracy and promotes interpretability using the model agnostic SHAP framework.

**Supplementary Information:**

The online version contains supplementary material available at 10.1038/s41598-024-75026-8.

## Introduction

Credit risk assessment is one of the main activities within the financial services industry, influencing the dynamics of lending and the broader economic landscape. Machine learning (ML) models have shown significant promise in enhancing predictive accuracy in credit risk assessment models because of their ability to model complex datasets. However, many credit risk analysts and regulators are wary of machine learning (ML) models because of concerns about their so-called “black box” nature. The black-box phenomenon alludes to the complexities of ML models that make interpreting their results challenging. In peer-to-peer (P2P) lending platforms, using black-box machine learning (ML) models to assess creditworthiness presents specific challenges. While efficient, these models lack transparency, making it difficult for borrowers and lenders to understand the decision-making process. This lack of interpretability in predictive ML models undermines trust in those models, especially in credit risk modelling and management, where decisions have significant financial consequences. In addition, this opacity challenges the adherence to fairness and regulatory compliance and hampers efforts to identify and mitigate biases that may disadvantage certain groups. Ultimately, black-box ML models lack transparency and accountability, undermining trust in the P2P platform and affecting its usability and acceptance. Unlike traditional linear regression models, where analysts can refer to the model’s estimated coefficients to interpret the model predictions, ML models are so complex that it is challenging to determine an established link between the model’s inputs and the output. A plethora of research attempts to address the trade-off between higher predictive power and explainablity, giving rise to the concept of eXplainable Artificial Intelligent (XAI)^1,2^. These explainable techniques focus on identifying the contributions of input features to the model’s overall and individual prediction.

This paper uses one of the frequently used XAI frameworks (Shapley Additive explanation), a concept borrowed from cooperative game theory, to open the “black box” of a novel ML-powered credit risk model^3^. The Shapley framework is a powerful tool for pinpointing sources of bias and facilitating more equitable lending practices by attributing the contribution of each feature to the prediction outcome. This study has three objectives: First, we compared the predictive accuracies of three distinct modelling approaches – a one-dimensional convolutional neural network $$\:\left(1DCNN\right)$$, $$\:XGBoost$$, a novel hybrid convolutional neural network and $$\:XGBoost$$$$\:(CNNs-XGBoost)$$ architecture and logistic regression $$\:\left(LR\right)$$ using a P2P consumer credit dataset with over twenty thousand observations. Second, we used the SHAP algorithm kernel-based explainers to explain the model’s ranking of overall features’ significance and provide interpretability^1^ of the basis of the model’s predictions. The comparative analysis allows us to assess the efficiency of these models and explore how advancements in XAI can bridge the gap between high predictive accuracy and the need for transparency in financial decision-making. Finally, we examined whether removing potentially discriminatory features, specifically gender and age, from the hybrid model would affect its ability to classify outcomes accurately.

In fulfilling our research objectives, this paper makes three original contributions to the existing body of knowledge and the credit risk management practice. First, we demonstrated a novel approach by enhancing the one-dimensional convolutional neural network $\:\left(1DCNN\right)$ performance through integration with an ensemble $\:XGBoost$ model using the generalised stacking framework. This contribution improves the literature by offering a hybrid model that outperforms traditional methods and provides a practical tool for credit risk analysts seeking more accurate risk assessments. Secondly, we addressed concerns regarding fairness and bias by demonstrating that eliminating potentially discriminatory features, such as age and gender, from the hybrid model does not significantly detract from its classification accuracy. This contribution is particularly relevant to the ongoing discourse on ethical AI, offering evidence that fair and unbiased credit scoring models do not have to compromise on effectiveness.

Thirdly, we showed that the $\:IDCNN\:$model better provided a complete view of the factors influencing credit risk than the $\:XGBoost$ because of the limitations of $\:XGBoost$ in providing local explainablity. Collectively, these contributions enhance our understanding of how to improve the accuracy, transparency, and fairness of ML-powered credit scoring models. The rest of the paper is structured as follows: Section two presents a brief review of related literature. In section three, we discussed the SHAP framework and the research hypothesis. Section four is the study’s methodology and description of the dataset, section five discusses the experiment’s results, and section six is the conclusion and direction for future research.

## Related studies

Credit risk assessment methodologies have evolved from traditional statistical techniques to sophisticated machine learning models. The transition to ML scoring models is exacerbated by the volume, variety and velocity of data available to banks, which have put them in a position to exploit advanced analytics in managing credit risk. Many studies have applied deep learning models in discriminating between good and bad credit risk. For example^4^, employed a hybrid method combining a relief algorithm with CNN to score credit risk. Their experiments revealed that the hybrid relief-CNN model showed better predictive accuracy than the logistic regression (LR) and random forest (RF) models. In another study^5^, used deep learning with clustering and merging, and the result showed high prediction accuracies. Similarly^6^, proposed a hybrid model using a dimension reduction approach and a multi-layer ensemble classifier. The results showed that the hybrid model achieved better performance in credit scoring. While^7^ compared ensemble methods and logistics regression in large dataset, and found that ensemble methods, particularly $\:XGBoost$ significantly outperformed logistic regression.

As^8^ highlighted, integrating Artificial Intelligence (AI) in credit scoring improves predictive accuracy but also raises concerns regarding transparency and interpretability. The opaque nature of these models limits the stakeholders’ ability to understand the basis of the model’s decision-making processes^9–11^. In response to these concerns, the field of Explainable AI (XAI) has emerged as a critical area of research, aiming to make AI systems more transparent and their decisions understandable to humans^2^. XAI is a set of methods that allows users to understand and trust the outputs created by machine learning methods. The most widely used XAI frameworks is the Shapley Additive exPlanation (SHAP) proposed by^3^. The SHAP is a model-agnostic XAI approach that can be applied to the predictive output, regardless of which model generated it^12^. It is based on the concept of cooperative game theory introduced by^13^. A unique feature of Shapley-based explainable method is its ability to explain single-point prediction (local explanations) and provide a global explanation- i.e. explaining the overall workings of the model (global explanations) in a fair and interpretable manner^12^.

Our study evaluates the contributions of loan customers’ age and gender to predicting default using three different ML models and highlights the important need to evaluate models based on their fairness and ability to reduce bias. This shift in focus emphasises the need to examine how well a model predicts default and how discriminatory features affects classification accuracy. The works of^14^ on one-dimensional Convolutional Neural Networks (1)^15^, on the $\:XGBoost$ model for corporate failure prediction, and^16^ on logistic regression provide a solid foundation for comparing the performance of these methods and the hybrid model in the context of credit risk assessment. Each study contributes to understanding the trade-offs between model complexity, performance, and interpretability.

## Shapley explainablity framework and formulation of hypothesis

Societies exhibit a wide range of biases and inequalities, which are inherently present in the data that train machine learning systems. Without deliberate efforts to counteract these biases, algorithms developed from such data will inevitably lead to prejudiced results, exacerbating the disparities that marginalised groups face^17^. Bias in machine learning models refers to systematic and unfair discrimination against certain groups based on data. Bias can occur due to various factors, including biased data collection, unrepresentative training datasets, or flawed algorithmic design^18^. For instance, ethnicity, age, gender, or socioeconomic biases can lead to unfair outcomes or decisions by automated systems^19^. Like all algorithmic decision-making systems, automated credit scoring systems are fed with historical loan data to predict future defaults. However, allowing discriminatory features such as ethnicity, gender, and age in the training dataset could result in a biased automated system. This potential bias can have significant consequences, affecting the fairness and accuracy of credit risk assessment. Consequently, removing these features from the credit scoring training dataset can reduce potential bias in such a system. However, the possible contributions of these features to the model’s classification accuracy are yet to be explored. The present study bridges that gap by introducing age and gender in the novel hybrid model to understand their sensitivity to the model’s classification accuracy. In addition, transparency in ML systems is one of the ways of reducing bias. Transparency in ML systems involves making the workings of ML systems understandable to humans, especially regarding how the model arrives at its decisions. The Shapley framework has been frequently used in previous studies to achieve transparency^8,12,20^.

### Shapley Value

The Shapley value, named after Lloyd Shapley, who introduced it in the 1950s, is a concept from cooperative game theory that offers a fair distribution of payoffs among players based on their contribution to the total payoff of the game^3^. The Shapley value is used in explainable artificial intelligence (XAI) to determine the importance of features in machine learning models. To understand the contribution of each feature to the model’s prediction, we calculate the Shapley values for all features^21^. Therefore, the SHAP value for any feature is the average marginal contribution of the feature across all possible feature coalitions.

To understand the mathematical foundation of Shapley values, consider a cooperative game with a set of players $\:N$, where each player is an integer from $\:1\:to\:n$. A coalition $\:S$ is any subset of $\:N$, including the empty set and $\:N$ itself. The game is defined by a characteristic function $\:\nu\::{2}^{N}\to\:\mathbb{R}$ that assigns a value to each coalition $\:S$, representing the total payoff that the members of $\:S$ can obtain by cooperating. The value of the empty set is Zero $\:\left(\nu\:\:\left(\varphi\:\right)=0\right)$. The Shapley value aims to distribute the total payoff generated by coalition $\:N$ among the players in a way that reflects their individual contributions. The contribution of a player $\:i$ to a coalition $\:S$ is the marginal value that $\:i$ adds to $\:S$, defined as $\:\upsilon\:\left(S\cup\:\left\{i\right\}\right)-\upsilon\:\left(S\right)$. The Shapley value of player $\:i,$ denoted by $\:{\varphi\:}_{i}\left(\upsilon\:\right)$, is calculated as the weighted average of $\:i{\prime\:}s$ marginal contributions to all possible coalitions that $\:i$ can join. Mathematically, it is given by:

1 $$\:{\varphi\:}_{i}\left(\upsilon\:\right)=\sum\:_{S\subseteq\:N\backslash\:\left\{i\right\}}\frac{\left|S\right|!\left(n-\left|S\right|-1\right)!}{n!}\left(\upsilon\:\left(S\cup\:\left\{i\right\}\right)-\upsilon\:\left(S\right)\right)$$

Where $\:\left|S\right|$ is the cardinality of set S, $\:n$ is the total number of players, and the summation is over all subsets $\:S\:$of $\:N$ that do not include player $\:i$. If we assume a linear model and we want to explain a prediction for a variable $\:{\varvec{x}}^{i}$. The prediction for $\:{\varvec{x}}^{i}$ is:

2 $$\:\widehat{\omega\:}\left({\varvec{x}}^{\left(i\right)}\right)=\alpha\:+{\beta\:}_{1}{x}_{1}^{\left(i\right)}+\dots\:+{\beta\:}_{p}{x}_{d}^{\left(i\right)}$$

Where $\:{x}_{1}^{\left(i\right)},{x}_{2}^{\left(i\right)},\dots\:,{x}_{d}^{\left(i\right)}$ are feature values. Let $\:{\varnothing\:}_{j}$ be a contribution of feature $\:j$ on prediction $\:\widehat{\omega\:}\left({\varvec{x}}^{\left(i\right)}\right)$. The contribution $\:{\varnothing\:}_{j}$ is given as:

$$\:{\varnothing\:}_{j}\left(\widehat{\omega\:}\right)={\beta\:}_{j}{x}_{j}^{\left(i\right)}-E\left({\beta\:}_{J}{\varvec{X}}_{j}\right)$$

3 $$\:={\beta\:}_{j}{x}_{j}^{\left(i\right)}-{\beta\:}_{j}E\left({\varvec{X}}_{j}\right),$$

Where $\:E\left({\varvec{X}}_{j}\right)$ is the expected value for feature $\:j$. If all feature contributions are summed up for one feature, the result is

$$\:\sum\:_{j=1}^{d}{\varphi\:}_{j}\left(\widehat{\omega\:}\right)=\sum\:_{j=1}^{d}\left({\beta\:}_{j}{x}_{j}^{\left(i\right)}-E\left({\beta\:}_{j}{\varvec{X}}_{j}\right)\right)$$

$$\:=\left(\alpha\:+\sum\:_{j=1}^{d}{\beta\:}_{j}{x}_{j}^{\left(i\right)}\right)-\left(\alpha\:+\sum\:_{j=1}^{d}E\left({\beta\:}_{j}{\varvec{X}}_{j}\right)\right)$$

4 $$\:=\widehat{\omega\:}\left({x}^{\left(i\right)}\right)-E\left(\widehat{\omega\:}\left(\varvec{X}\right)\right),$$

Equation (4) is the difference between the predicted value for the feature $\:{x}^{\left(i\right)}$ and the average predicted value for the dataset $\:\varvec{X}={\left\{{\varvec{X}}_{j}\right\}}_{j}^{d}=1$ where $\:{\varvec{X}}_{j}={\left\{{x}_{ij}\right\}}_{i}^{N}=1$.

The Shapley framework has several desirable properties that justify its widespread use in XAI studies. First is its efficiency, which ensures that the total payoff generated by a cooperative game is fully distributed among the players. The second property is the symmetry property, which implies that if two features contribute equally to the prediction outcome across all possible subsets of features, they should be assigned the same importance or Shapley value.

The symmetry principle ensures that the Shapley value does not discriminate between players based on their identity but rather on their contribution to the game. This property underpins the fairness and equity of the Shapley approach in attributing contributions in explainable AI, ensuring that identical contributions receive identical rewards. The third property is the dummy player property which plays a critical role in explainable AI by helping to identify and disregard non-contributing features in predictive models. Suppose a feature is determined to be a dummy (i.e., its inclusion or exclusion does not change the model’s predictions), in that case, its Shapley value will be low or zero, indicating little to no importance. This enhances the interpretability and efficiency of models and ensures that explanations are focused on features that genuinely influence the model’s predictions. The dummy player property respects the principle of fairness in the model explanation. It prevents overestimating the importance of features that, in reality, do not influence the model’s decisions. The above-mentioned Shapley properties help in enhancing transparency in ML models.

In this study, we addressed the biases in ML credit scoring systems by proposing and evaluating a hybrid model through various hypotheses. First, we assessed the effectiveness of the hybrid model’s performance in credit risk analysis. The null hypothesis ($\:{H}_{0}$) is that the hybrid model does not significantly improve the accuracy of credit risk predictions compared to conventional credit scoring models. While the alternative hypothesis $\:\left({H}_{1}\right)$ is that the hybrid model significantly improves the accuracy of credit risk predictions compared to traditional credit scoring models.

5 $$\:{H}_{0}:{\mu\:}_{{\Omega\:}}={\mu\:}_{LR};{\mu\:}_{CNN};{\mu\:}_{XGBoost}$$

6 $$\:{H}_{1}:{\mu\:}_{{\Omega\:}}\ne\:{\mu\:}_{LR};{\mu\:}_{CNN};{\mu\:}_{XGBoost}$$

Where $\:{\mu\:}_{{\Omega\:}}$ represents the mean predictive accuracy of the hybrid model. $\:{\mu\:}_{LR};{\mu\:}_{CNN};{\mu\:}_{XGBoost}$ represents the mean predictive accuracies of the logistics regression, the one-dimensional neural network and the $\:XGBoost$. Second, we explored the explainablity of decisions made by the one-dimensional $\:1DCNN$ model compared to logistic regression and $\:XGBoost$. Here, $\:{H}_{0}$ suggests that there is no significant difference in the explainablity of credit risk decisions between the $\:1DCNN$ model and traditional methods ($\:XGBoost\:and\:LR$). While the alternative hypothesis is that the explainablity of the credit risk decisions made by the $\:1DCNN$ model is significantly better than those made by traditional credit scoring models. Let’s denote the explainablity of the $\:IDCNN$ model as $\:{E}_{1D}$ and the explainablity of traditional models as $\:{E}_{trad}$.

7 $$\:{H}_{0}:{E}_{1D}-{E}_{trad}=0$$

8 $$\:{H}_{1}:{E}_{1D}-{E}_{trad}>0$$

Where $\:{E}_{1D}$ and $\:{E}_{trad}$ are measured using the contributions of the inputs on the model’s decision. Lastly, we investigated the effect of excluding discriminatory features on the hybrid model’s accuracy. Let us denote the classification accuracy of the hybrid model with all features as $\:{A}_{Full}$ and the model’s classification accuracy after removing discriminatory features as $\:{A}_{Reduced}$. Mathematically, these hypotheses can be represented as:

9 $$\:{H}_{0}:{A}_{full}-{A}_{reduced}=0$$

10 $$\:{H}_{1}:\:{A}_{full}-{A}_{reduced}\ne\:0$$

In this formulation, $\:{A}_{full}$ represents the accuracy of the model when it includes all features, and $\:{A}_{reduced}$ represents the accuracy after the removal of discriminatory features. The null hypothesis $\:{H}_{0}$ suggests that the accuracy remains unchanged after removing such features (age and gender). In contrast, the alternative hypothesis $\:{H}_{1}$ suggests a significant difference in accuracy, indicating that the discriminatory features had an impact on the model’s performance.

## Methodology and data

**Fig. 1:**
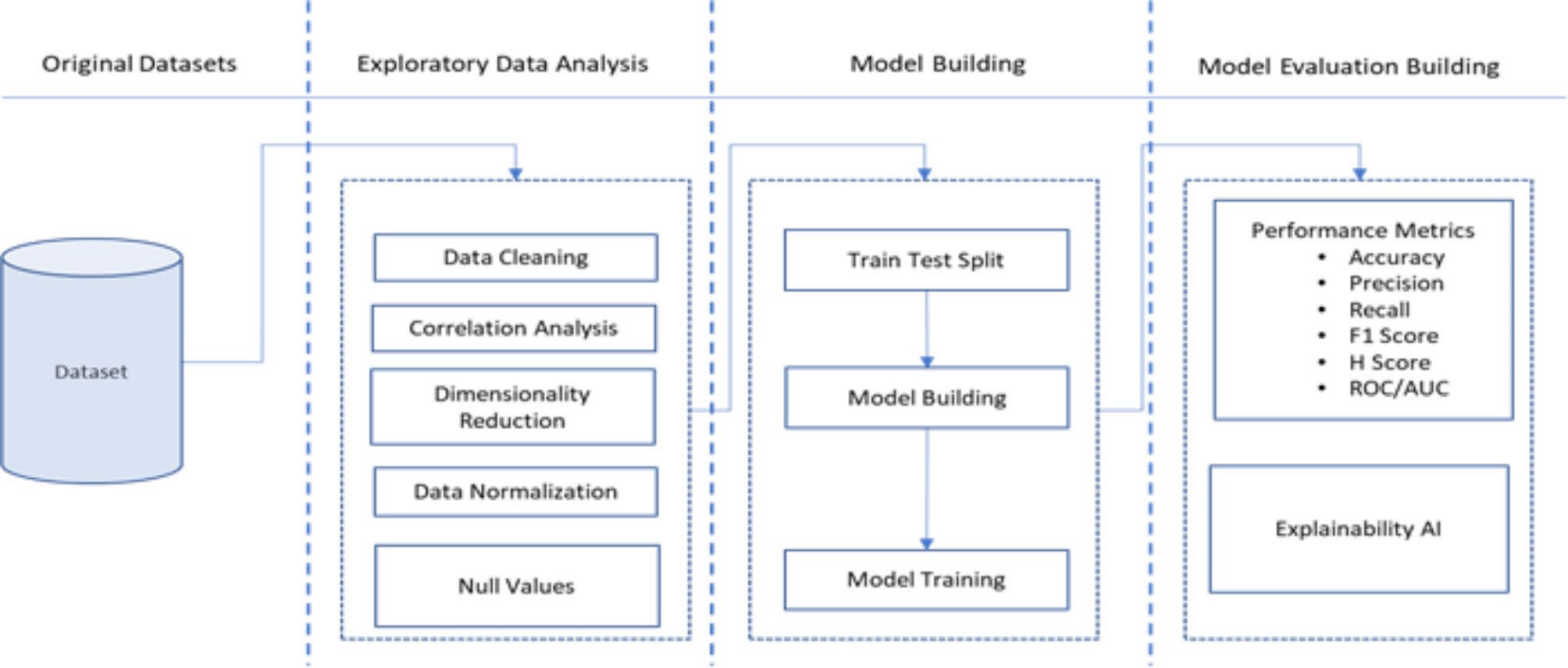
Process flow diagram of experiment methodology.

We employed a hybrid machine learning model that combines a 1DCNN with XGBoost using a generalised stacking framework and the SHAP algorithm for feature importance analysis. The software tool used is Python Programming Language, which runs on Jupiter Notebook. The Python packages used include Keras, Sklearn, NumPy, Pandas, Shap, Matplotlib, and XGBoost Classifier, with additional reliance on Python-based libraries for data pre-processing and model evaluation. Figure 1 shows a schematic diagram of the experiment’s process flow. This involves three iterative processes: exploratory data analysis, model building, and model evaluation.

The dataset used in this experiment was customer loans from Lending Club, a P2P lending company. The original dataset is publicly available on^22^ and corresponds to all the loans issued by the firm between 2007 and 2018. The dataset has one million forty-eight thousand five hundred and seventy-six observations with one hundred and forty-five input features. During the exploratory data analysis, we removed features with more than 40% missing values and dropped highly correlated features. The reduced input feature space used for this experiment comprises twenty-five features and twenty-five thousand, five hundred and thirty-five observations. Next, we addressed labelling categorical variables, transforming them into a numerical format using label encoding. We used the Min-Max scaling technique to normalise the input features, which scales the features to a fixed range of 0 to 1. The formula used is:

11 $$\:{X}^{{\prime\:}}=\frac{X-\text{m}\text{i}\text{n}\left(X\right)}{MAX\left(X\right)-\text{m}\text{i}\text{n}\left(X\right)}$$

For the training phase, the dataset was split into training (80%) and testing (20%) samples to provide a robust evaluation of the model’s performance on unseen data. The 80/20 split is a commonly used rule in machine learning for dividing data into training and testing sets^8,20^. This ratio achieves a sufficient balance between having enough data to train the model effectively (80% for training) and enough data to test and evaluate the model’s performance (20% for testing). With larger datasets, the 80% training portion would capture the variability and distribution of features within the data, which means the model training covers most scenarios expected in the application. We adopted the $\:K-fold\:$validation method to assess the effectiveness of the models, particularly to avoid overfitting. This approach randomly divides the dataset into $\:K\:groups$ or approximately equal-sized folds^23^. The model is trained $\:K\:times$, using $\:K-1$ folds as the training set and the remaining single fold as the validation set. This process is repeated so that each of the $\:K\:folds$ is used as the validation set exactly once.

This study used 5 groups (*5-fold cross-validation*). The first 4-subsets were used to develop the training sample for the model. The resulting model is assessed for accuracy on the remaining $\:1/5th$ of the sample (test sample). The results of the models from each fold are averaged to produce a single estimation. This averaging helps to reduce variability and provides a more accurate measure of model performance. Finally, we employed Grid Search as our optimisation strategy to select the optimal hyperparameters for each model. This method works through all possible combinations of parameters, which, while potentially computationally expensive, ensures that no potential configuration within the specified range is overlooked^24,25^. This can be particularly advantageous in scenarios where the optimal hyperparameter values are unknown, and a detailed search is necessary to ascertain the best-performing parameters.

### Logistic regression (LR)

(LR) is a statistical method that analyses the relationship between one or more independent variables and a binary dependent variable^26^. logistic regression is used for binary classification tasks, making it highly relevant in credit risk analysis, where the goal is to predict whether an event (loan default) will occur^16^. The core of logistic regression is the logistic function (Sigmoid function), which maps any real number into a value between 0 and 1, making it interpretable as a probability. The logistic function is defined as:

12 $$\:P\left(Y=1\right)=\frac{1}{1+{e}^{\left({\beta\:}_{0}+{\beta\:}_{1}{X}_{1}+{\beta\:}_{2}{X}_{2}+\dots\:+{\beta\:}_{n}{X}_{n}\right)}}$$

Where, $\:P\left(Y=1\right)$ represents the probability of the dependent variable equals to 1 (loan default), $\:e$ is the base of the natural logarithm, $\:{\beta\:}_{0}$ is the intercept term, and $\:{\beta\:}_{1}$, $\:{\beta\:}_{2}$,…,$\:{\beta\:}_{n}$ are the coefficients of the independent variables $\:{X}_{1}$, $\:{X}_{2}$,…,$\:{X}_{n}$, which influence the outcome.

13 $$\:Odds=\frac{P\left(Y=1\right)}{1-P\left(Y=1\right)}$$

Equation (13) is the odds ratio which is the ratio of the probability of the event occurring to the probability of it not occurring.

14 $$\:log\left(\frac{P\left(Y=1\right)}{1-P\left(Y=1\right)}\right)={\beta\:}_{0}+{\beta\:}_{1}{X}_{1}+{\beta\:}_{2}{X}_{2}+\dots\:+{\beta\:}_{n}{X}_{n}$$

Equ (14) is the natural logarithm of the odds ratio which leads to the logit function. The equation linearly relates the independent variables to the log odds of the dependent event. While logistic regression provides a straightforward, interpretable model for credit risk prediction, its linear nature and limitations in handling complex, non-linear interactions and unstructured data make it less effective in capturing the full spectrum of risk factors.

#### XGBoost

$\:XGBoost$ builds on the gradient boosting framework, where new models are added sequentially to correct the errors made by existing models^27^. The main idea is to consecutively add trees to the model, where each new tree corrects the residual errors made by the existing ensemble of trees. The overall prediction model in $\:XGBoost$ is an ensemble of decision trees formulated as:

15 $$\:{\widehat{y}}_{i}={\sum\:}_{k=1}^{K}{f}_{k}\left({x}_{i}\right)$$

Where $\:{\widehat{y}}_{i}$ is the model’s prediction for the $\:{i}^{th}$ instance, $\:{f}_{k}$represents an individual decision tree, $\:{x}_{i}$ is the feature vector for the $\:{i}^{th}$ instance, and $\:\text{{\rm\:K}}$ is the number of trees. The objective function in $\:XGBoost$ combines a loss function $\:L$ which measures how well the model’s prediction match the actual data. The regularisation term $\:{\Omega\:}$ penalises the complexity of the model to prevent overfitting. It can be expressed as:

16 $$\:Obj={\sum\:}_{i}L\left({y}_{i},{\widehat{y}}_{i}\right)+{\sum\:}_{k}{\Omega\:}\left({f}_{k}\right)$$

Where, $\:{y}_{i}\:$is the actual value for the $\:{i}^{th}$ instance, and $\:{\Omega\:}\left({f}_{k}\right)$ represents the regularisation term for the $\:{k}^{th}$ tree. $\:XGBoost$ optimises this objective function using a gradient descent approach, where it calculates the gradient of the loss function with respect to the model’s predictions. The model is updated in a direction that minimises the loss. The regularisation term helps in reducing overfitting by controlling the model’s complexity. Its gradient boosting mechanism enables the model to learn complex, non-linear relationships between the features and the target variable, which is crucial for accurately assessing credit default risk. Although $\:XGBoost$ offers several advantages for predicting credit risk, it has limitations that necessitate using our proposed hybrid model. A significant limitation of this model is its approach to sequential or temporal data. $\:XGBoost$ is designed to handle static, structured data like most tree-based models. Sequential or temporal data contains dependencies where the significance of a current observation may depend on previous observations. For example, a series of missed payments or a steadily increasing debt-to-income ratio over time could indicate a growing risk of default that might not be as evident when considering these factors independently of their temporal context. $\:XGBoost{\prime\:}s$ standard approach does not explicitly model such temporal dependencies, which could lead to less accurate risk assessments for individuals with significant behavioural patterns over time.

#### Convolutional neural network

**Fig. 2:**
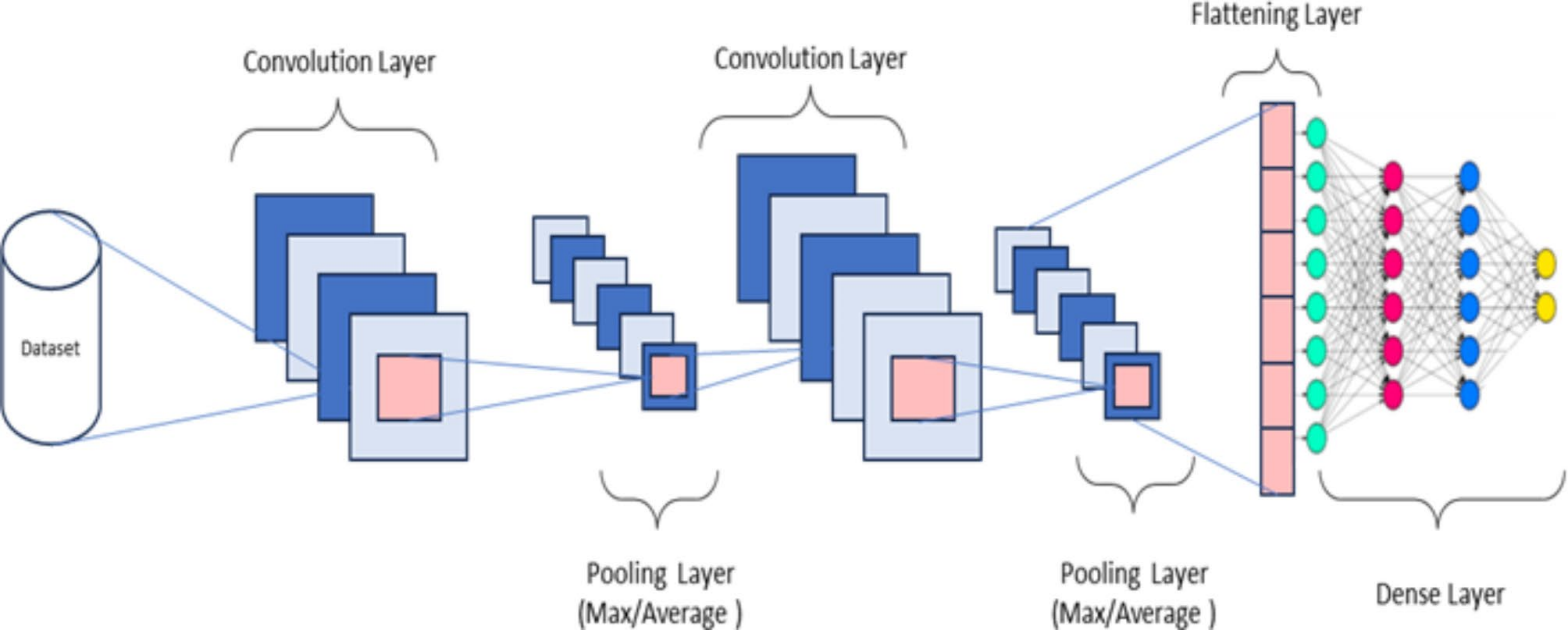
Schematic diagram of a convolutional neural network.

Convolutional neural network (CNN) offers an optimal architecture for learning complex features in large datasets heuristically. CNN has significant applications in computer vision, image classification/analysis^28^, nuclear and particle physics^29^. A key feature of CNN methods is their ability to achieve ‘spatial invariance’-i.e. they can learn to recognise and extract image features anywhere in the image (Ibid). CNN uses a unique feature called ‘convolution’ to condense images to a form that is easier to process without losing features crucial for prediction accuracy. Figure 2 below is a schematic diagram of a CNN. It shows the flow from the input layer through convolutional layers, followed by pooling layers, to a flattening step. After flattening, the data passes through dense (fully connected) layers, culminating in an output layer with a sigmoid activation function for the binary classification. The convolutional layer applies a set of learnable filters to the input data. In the context of a $\:1DCNN$, these filters slide (convolve) over one dimension of the data, processing one-dimensional sequence data. This operation aims to extract high-level features from the input sequence. Each filter in the convolutional layer is designed to detect specific features, such as trends. The output of this layer is a set of feature maps representing the presence of detected features across the sequence.

A non-linear function such as the Rectified Linear Unit (ReLU) function is applied on feature maps. In a binary classification task, the last dense layer has a single neuron with a sigmoid activation function, which outputs a probability indicating the likelihood of the input belonging to one class or the other. The one-dimensional convolutional neural network $\:\left(1DCNN\right)$ is capable of analysing temporal and sequential data, making it an ideal tool for examining financial behaviours over time. Unlike traditional neural networks and ensemble models, $\:1DCNN$ applies convolutional operations to one-dimensional data, extracting significant features from sequences such as payment histories or credit utilisation trends. This capability allows the model to identify temporal patterns that signal potential financial distress or stability in borrowers, providing critical insights for predicting loan defaults. Although CNNs are very effective in local and hierarchical pattern detection in data, they might only partially capture complex, non-linear interactions between features across the whole dataset. XGBoost can complement this by explicitly modelling these interactions and dependencies. Hence, there is a need to develop a hybrid model that leverages the strengths of both models.

#### The hybrid model

Our hybrid model employs a specialised technique known as generalised stacking, which layers multiple initial models to form a composite, hybrid architecture. The generalised stacking method is better suited for hybridisation because it allows the meta-model (XGBoost) to learn from the residuals or errors of the base models’ (CNNs) initial predictions. This process refines the final output by addressing and correcting specific weaknesses in the base models’ predictions. In addition, the generalised stacking offers flexibility in how the models are combined. For instance, the meta-model was trained to focus on aspects where the base models underperform, such as overfitting training data, leading to poor generalisation of unseen data. The meta-model (XGBoost) mitigates this by adjusting the final decision boundary based on a detailed understanding of errors made by the CNNs.

Our objective in introducing the hybrid model is to enhance features extraction and robustness by integrating diverse modelling approaches. The hybrid model capitalises on the complementary strengths of deep learning and ensemble techniques. Specifically, the one-dimensional CNNs can effectively capture local dependencies and patterns in the data, leveraging their strength in handling sequence or time-series data. On the other hand, XGBoost is very effective in handling structured data and can model the non-linear interactions between credit risk features. Overall, the hybrid model can better generalise to new data by taking advantage of the strengths of each model, reducing the risk of overfitting to the training data compared to using a single model.

**Fig. 3:**
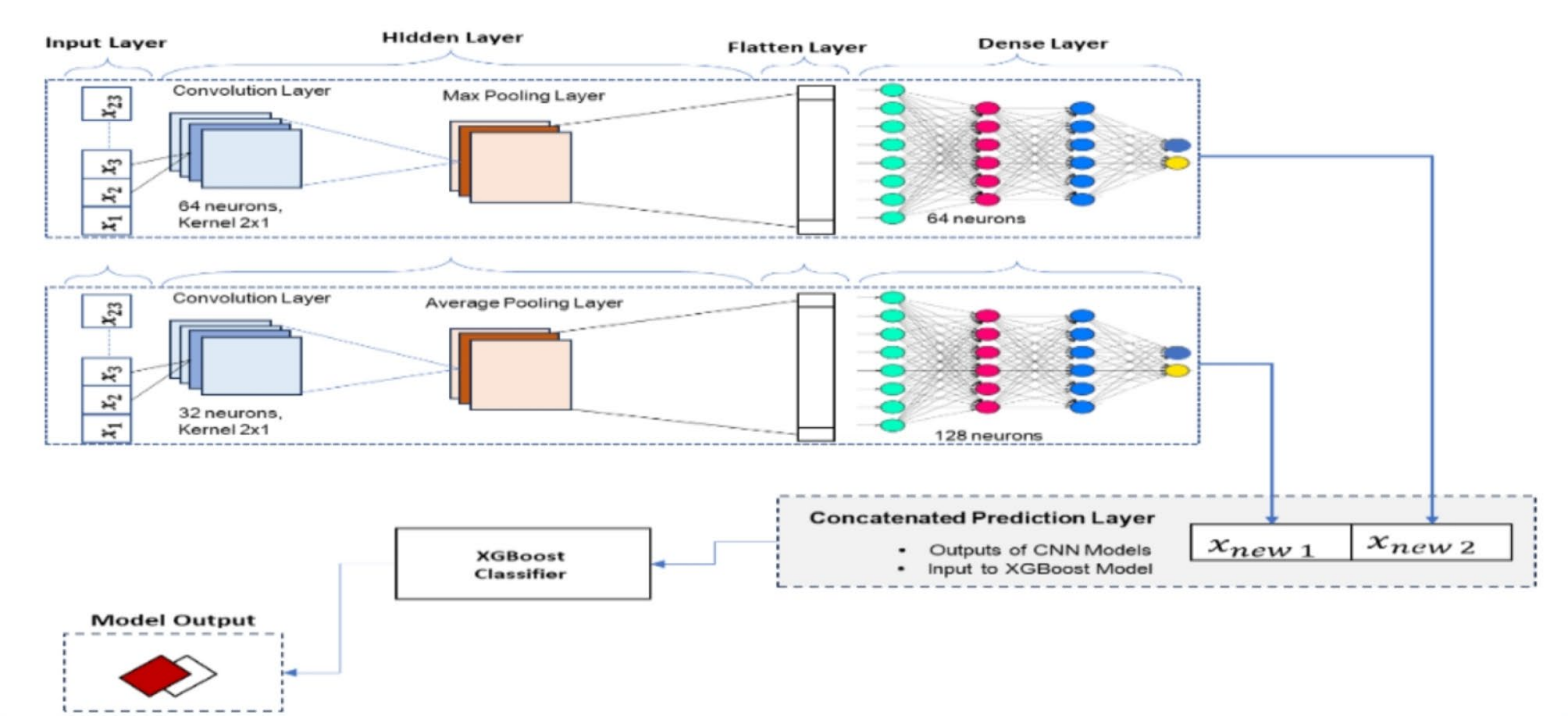
Architecture of the hybrid $\:CNN-XGBoost$ model.

Figure 3 is the schematic diagram of the hybrid architecture. Specifically, the dataset was trained using 1DCNNs. The predictions from these base models are used as input features for another model (meta model)—$\:XGBoos$. After the data pre-processing discussed in Sect. Methodology and data, we designed the architectures for the two one-dimensional CNNs. We trained the data independently to capture different patterns in the data, with their predictions forming meta-features. These meta-features create a new dataset for training the meta-model. XGBoost is trained on this meta-dataset to learn from and correct errors made by the CNNs, optimising performance through hyperparameter tuning. The stacked model’s predictions are validated using the performance evaluation metrics discussed in Sect. Performance evaluation metrics. The hybridisation takes advantage of the CNN models for feature extraction capabilities and XGBoost for handling complex feature interactions.

#### Data

**Table 1:** Descriptive statistics of the features.

Feature name	Mean	Standard deviation	Maximum	Minimum
$\:Emp\_Length$	5 years	4 years	11 years	0 year
$$\:Annual\_Inc$$	$80,696.06	$81,295.23	$5,119,032.00	$0.00
$\:Debt\:to\:income\:DTI$ $\:ratio$	19.67	17.09	25.57	0
$\:Age$	49 years	18 years	80 years	33 years
$$\:Loan\_amt$$	$16,618.45	$10366.70	$40,000	$1000
$\:Term$	44 months	11 months	60 months	36 months
$\:Interest\:rate$	14.54%	5.56%	30.99%	5.31%
$$\:Total\:bankcard\:Credit\:limit$$	$25,476.43	$25,594.13	$4,174,00.00	$0.00
$$\:Revolving\:line\:utilization$$	$43,531.28	$25,374.95	$146,300	$0.00
$$\:Total\:credit\:balance\:excluding\:mortgage$$	$52,594.60	$54,106.31	$1,077617.00	$0.00
$$\:Revolving\:balance$$	$15,710.89	$22,833.64	$1,049,095.00	$0.00
$$\:Total\:payments$$	$1785.03	$2522.43	$40,856.679	$0.00
$$\:Remaining\:outstanding\:principal$$	$12,288.02	$10,830.76	$40,000.00	$0.00
$$\:Interest\:received\:to\:date$$	$629.27	$763.65	$8898.69	$0.00
$$\:Total\:current\:balance\:of\:all\:account$$	$137,766.00	$163,096.10	$216,3151.00	$0.00

**Fig. 4:**
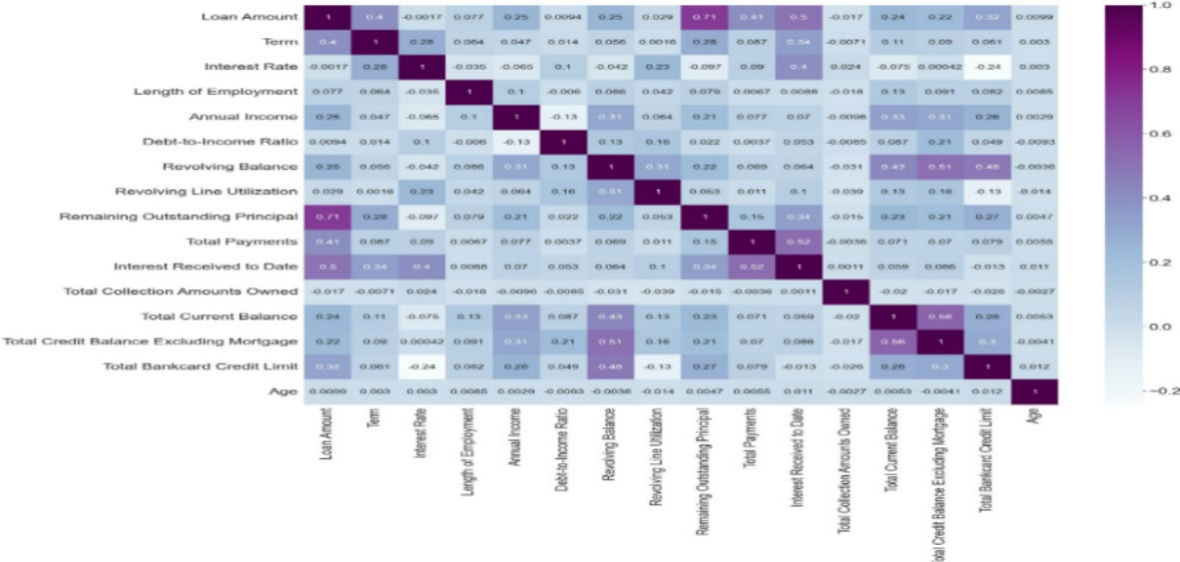
Correlation matrix.

Table 1 is the descriptive statistics of the numeric features. Using Pearson’s correlational heat map on continuous variables and the $\:Chi-square$ test for categorical variables, we identified and dropped highly-correlated variables. We experimented with the new credit risk dataset containing a reduced dimension in the input feature space. The features cover a range of information pertinent to assessing the risk and viability of the loans from both the borrower’s financial and personal standpoint. Based on the distribution of the dataset, we created two demographic data, gender and age, to test our second hypothesis. Figure 4 shows the correlation heatmap. Some interesting insights from the correlation suggest a strong positive correlation of 71% between the loan amount and outstanding principal, indicating that higher loan amounts tend to have higher remaining principals.

This could be expected as larger loans take longer to pay off. A moderate positive correlation of 28% between interest rate and loan term suggests that longer-term loans come with higher interest rates. We discovered a significant negative correlation between loan status and interest rate. This suggests that higher interest rates are associated with a specific loan status outcome. This could imply that loans with higher interest rates are less likely to be in good standing, perhaps due to the increased financial burden on the borrower. There was a negative correlation between loan status and annual income, indicating that the probability of a loan default decreases as the borrower’s annual income increases. We consider these relationships within the broader context of loan management and borrower behaviour to glean insights into risk factors and opportunities for improving loan outcomes. Table 6 in the appendix presents the definitions and measurements of the variables employed in our study.

#### Performance evaluation metrics

We used the following metrics to test the performance and predictive accuracies of the $\:1DCNN$, $\:XGBoost$, logistic regression and the hybrid models. Precision (P) is a metric that measures the number of correct (True) positives classifications. It is calculated as the ratio of correctly predicted positive (True positives) and all the total number of positives that were predicted.

17 $$\:Precision=\frac{True\:Positive\:\left(TP\right)}{True\:Positive\:\left(TP\right)+False\:Positive\:\left(FP\right)}$$

The Recall which measures the number of True positive classification made out of all positive predictions is another evaluation metric used in this paper. It measures the model’s ability to correctly identify True Positives and is measured as

18 $$\:Recall=\frac{True\:Positive\:\left(TP\right)}{True\:Positive\:\left(TP\right)+False\:Negative\:\left(FN\right)}$$

The F-1 score combines the precision and recall scores of the model to measure the model’s accuracy. This evaluation metric is calculated as the harmonic mean of the precision.

and recall scores and it is calculated as:

19 $$\:F1-score=2\times\:\frac{Precision\times\:Recall}{Precision+Recall}$$

The precision and recall for each class (default and non-default) were used to calculate the macro and weighted scores using the formulae

20 $$\:macro-average=\:\frac{{\sum\:}_{k}{SCORE}_{k}}{N}$$

21 $$\:weighted-average={\sum\:}_{k}{SCORE}_{k}{W}_{k}$$

The $\:{SCORE}_{k}$ in Eqs. 19 and 20 denote either the precision, recall and F1-score for each of the target variable class $\:k=\left\{0\:and\:1\right\}$ and $\:{W}_{k}$ is the ratio of the number of examples in the training or testing dataset. We also calculated the area under the curve (AUC) and the $\:H-measure$ for the hybrid. The AUC measures the predictive power of a model^21^ and it is calculated as follows:

22 $$\:AUC=\frac{1}{2}\left(1+\frac{TP}{TP+FN}-\frac{FP}{FP+TN}\right)$$

The higher the AUC, the better the predictive power of the model. To measure the cost of misclassification in our hybrid model, we calculated the $\:H-measure$, a statistical measure proposed by Hand in 2009 as an alternative to the ROC curve (AUC) used for evaluating the performance of binary classification models^30^. argues that the AUC does not consider that different misclassification errors can have different consequences. Consequently, the $\:H-measure$ addresses this limitation by incorporating the concept of misclassification costs into its calculation. It acknowledges that the costs associated with false positives and negatives can vary greatly depending on the application. In the current case, the cost of mistakenly granting a loan to a customer who defaults (false positive) often differs from the cost of denying a loan to a customer who would not have defaulted (false negative). The $\:H-measure$ is calculated as:

23 $$\:H=1-\frac{EC\left({hb}_{classifier}\right)}{EC\left({rd}_{classifier}\right)}$$

Where $\:EC\left({hb}_{classifier}\right)$ is the expected cost of the hybrid classifier and $\:EC\left({rd}_{classifier}\right)$ is the expected cost of a random classifier. The measure ranges for 0 to 1, where 1 indicates no misclassification cost and 0 indicates a performance equivalent to random guessing.

## Discussion of results from the experiment

Table 2 below presents the results for our first hypothesis, which tests the effectiveness of hybrid model, the $\:1DCNN$, logistic regression and $\:XGBoost$ models in predicting default. The null hypothesis is that the hybrid model does not significantly improve the accuracy of credit risk predictions compared to traditional credit scoring models. We used the macro and weighted average scores to summarise individual model performances across all classes. While the macro average assesses the model’s ability to predict each class with equal importance to ensure the model performs well across all classes, including the minority ones. Conversely, the weighted average calculates the metric for each class, weighting it by the class’s prevalence or support (the number of true instances for each class). Table 2 suggests that the two metrics are the same for the $\:1DCNN$ and the hybrid model − **0.96**. However, the classification accuracy for the models indicates that the hybrid model slightly outperforms the conventional models, rejecting the null hypothesis of no improvement in classification accuracy with the hybrid approach. Specifically, the hybrid model achieved a classification accuracy of **96%**, compared to **94%** for the $\:1DCNN$, **94%** for the $\:XGBoost$ and **92%** for the logistic regression model.

This 2% enhancement in classification accuracy with the hybrid model is significant in the context of credit risk assessment. In such models, a 2% increase in accuracy can significantly impact the precision of risk evaluation, potentially leading to more reliable identification of defaulters versus non-defaulters. This improvement directly translates to reduced financial risk and losses for lending institutions by enabling more informed lending decisions, highlighting the value of even seemingly modest gains in model performance.

**Table 2:** Performance evaluation metrics across the different ML models with all the variables. $\:{H}_{0}:{\mu\:}_{{\Omega\:}}={\mu\:}_{LR};{\mu\:}_{CNN}$

Hybrid *1DCNNs-XGBoost*						
	Precision	Recall	F1-Score	$\:Accuracy$	$\:AUC$	$\:H-Score$
0	0.94	0.98	0.96	**0.96**	**0.98**	**0.95**
1	0.98	0.94	0.95			
$\:Macro\:Avg.$	0.96	0.96	0.96			
$\:Weighted\:Avg$	0.96	0.96	0.96			
$$\:1\varvec{D}\varvec{C}\varvec{N}\varvec{N}$$						
0	0.95	0.99	0.97	0.94	0.98	0.92
1	0.98	0.94	0.96			
$\:Macro\:Avg$	0.96	0.96	0.94			
$\:Weighted\:Avg$	0.96	0.96	0.96			
$$\:\varvec{L}\varvec{o}\varvec{g}\varvec{i}\varvec{s}\varvec{t}\varvec{i}\varvec{c}\varvec{s}\:\varvec{R}\varvec{e}\varvec{g}\varvec{r}\varvec{e}\varvec{s}\varvec{s}\varvec{i}\varvec{o}\varvec{n}$$						
0	0.88	0.98	0.93	0.92	0.95	0.95
1	0.98	0.86	0.92			
$\:Macro\:Avg$	0.93	0.92	0.92			
$\:Weighted\:Avg$	0.93	0.92	0.92			
$$\:\varvec{X}\varvec{G}\varvec{B}\varvec{o}\varvec{o}\varvec{s}\varvec{t}$$						
0	0.92	0.98	0.95	0.94	0.99	0.95
1	0.98	0.91	0.94			
$\:Macro\:Avg$	0.95	0.94	0.94			
Weighted Avg	0.95	0.94	0.94			

Additionally, the improvements in performance metrics, detailed in Table 2, support the decision to combine CNN and XGBoost into a hybrid model. We saw improvements in classification accuracy (0.98 vs. 0.94), precision for the non-default class (0.94 vs. 0.92), recall for the default class (0.94 vs. 0.91), and F-score for both classes. This synergy improves the overall accuracy and enhances the model’s ability to generalise across diverse datasets.

**Fig. 5:**
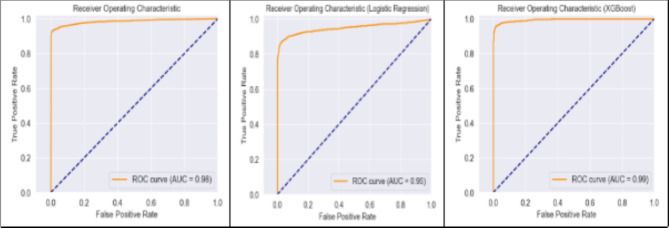
AUCs for the hybrid, LR and $\:XGBoost$ models.

Figure 5 above shows the AUC scores for the three models. We found the AUC curve for the $\:XGBoost$ model is slightly higher at (0.99), than the Hybrid (0.98) and the logistic regression (0.95). The $\:H-score$, which adjusts for the distribution of class probabilities and the cost of misclassifications, being 0.95 for all the models except the 1DCNN suggest that the models are highly effective at balancing the trade-offs between false positives and false negatives. This indicates that the models predict accurately and does so in a way that align with the practical cost considerations of misclassifying instances. Therefore, we can conclude that the hybrid model effectively differentiates between clients who will default and those who will not, with a strong emphasis on minimising costly errors (like incorrectly identifying a good client as a risk or vice versa).

**Table 3:** Comparison of the hybrid model and other models using the German credit dataset.

	Accuracy	Precision
$\:XGBoost\:$classification	80%	76.46%
Support Vector classification	76%	85.19%
Random Forest Classification	83.2%	82.95%
Neural Network Classification	70.4%	65.18%
Logistic Regression	80.8%	78.04%
Hybrid Model	**90.0%**	**84%**

**Source**: Hoffman (1984) Statlog (German Credit Data). Baseline Performance and hybrid model performance.

To evaluate the effectiveness of the hybrid model, we applied it to the German credit dataset, originally used by Hofmann in 1994, allowing us to benchmark its classification accuracy against the results from other studies that also utilised this dataset. Table 3 shows the performance evaluation for the baseline models, as reported by^31^, and the performance of the hybrid model. We assessed model performance using accuracy and precision as metrics. The results indicated that the hybrid model performed better than the traditional models on the German Credit dataset.

**Table 4:** Comparative performance of ML models on P2P lending datasets.

Authors	Dataset	State of the act studies	AUC	Accuracy
Zhang et al., (2020)^36^	Lending club P2P U.S. A	Online integrated credit scoring model (OICSM). OICSM integrates gradient boosting decision tree (GBDT) and neural network (NN).	0.72	
Ahelegbey and Giudici (2023)^37^	SMEs P2P lending across Europe	Factor clustering-based approach segmenting a heterogeneous population into groups with more homogeneous characteristics.	0.82	
Lyócsa et al., (2022)^38^	Lending club P2P U.S.A	Neural Network Classification	N/R	0.72
An-Hsing et al., (2022)^39^	Lending Club P2P U.S. A	2-Layer Neural network	N/R	0.87
Hybrid model	Lending club P2P U.S. A	Hybrid (*1DCNN-XGBoost*)	**0.98**	**0.96**

Note: Most of the included studies compared more than one ML model, and we reported the model with the highest AUC or accuracy scores.

Additionally, we assessed the performance of different ML models on the Peer-to-Peer (P2P) dataset and compared these results to the hybrid model’s performance, providing a contextual performance evaluation. Table 4 is the performance data from a selection of these studies. The proposed model demonstrated superior performance, achieving an AUC of 0.98 and an accuracy of 0.96. However, variations in the configurations of the P2P datasets might influence these performance metrics. For example, two studies using the Lending Club dataset could report different results due to significant differences in the selection of input features and the number of observations included in the analysis. We also explored the models’ explainablity- the second hypothesis. Using Global explainablity, we sought to understand how the $\:1DCNN$, $\:XGBoost$ and $\:LR$ models prioritise the importance of different features when assessing credit risk. The findings from our experiments provided clear evidence to reject the null hypothesis that all the models would similarly explain credit risk decisions.

**Fig. 6:**
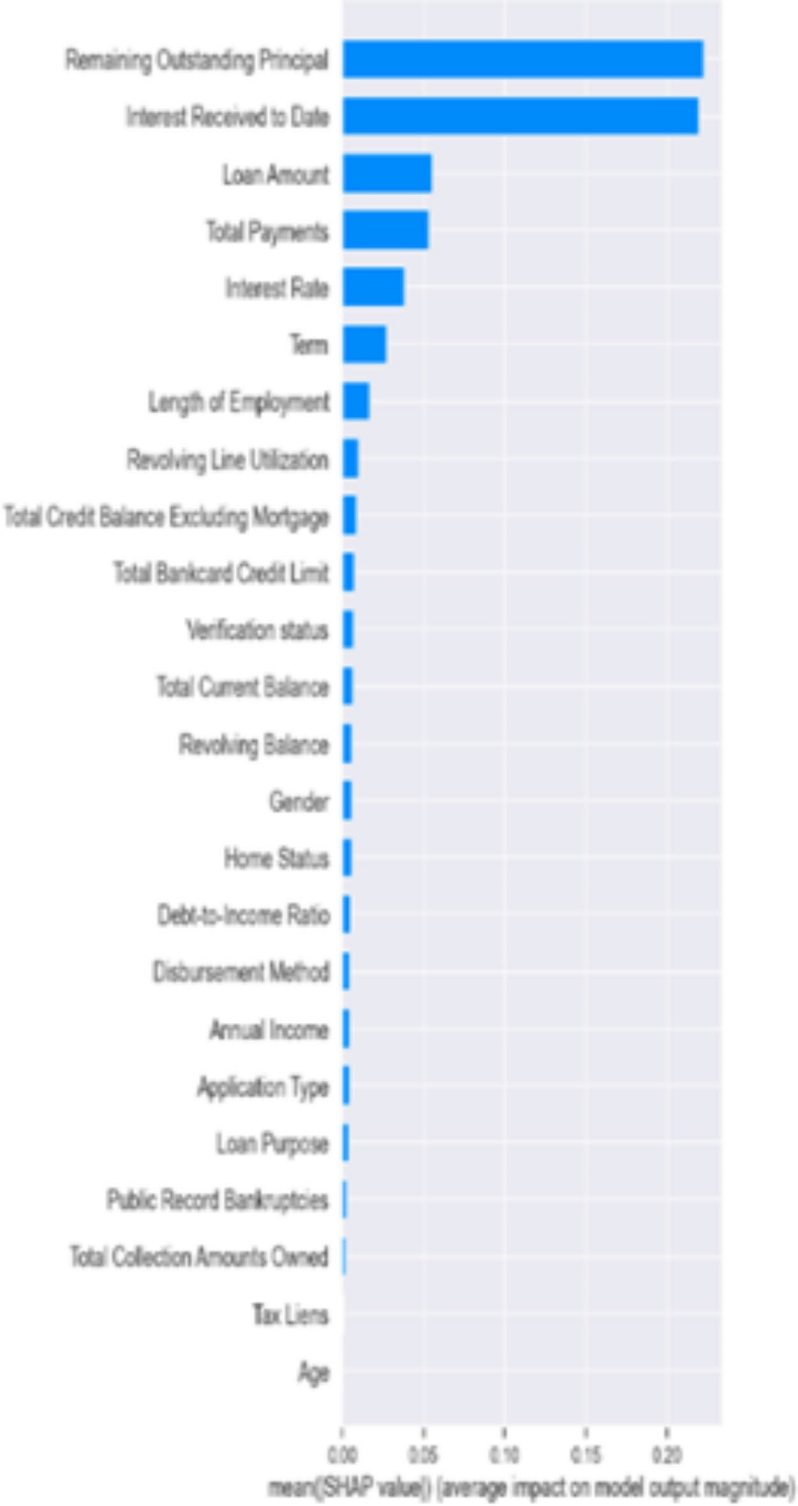
Features ranking 1DCNN model.

**Fig. 7:**
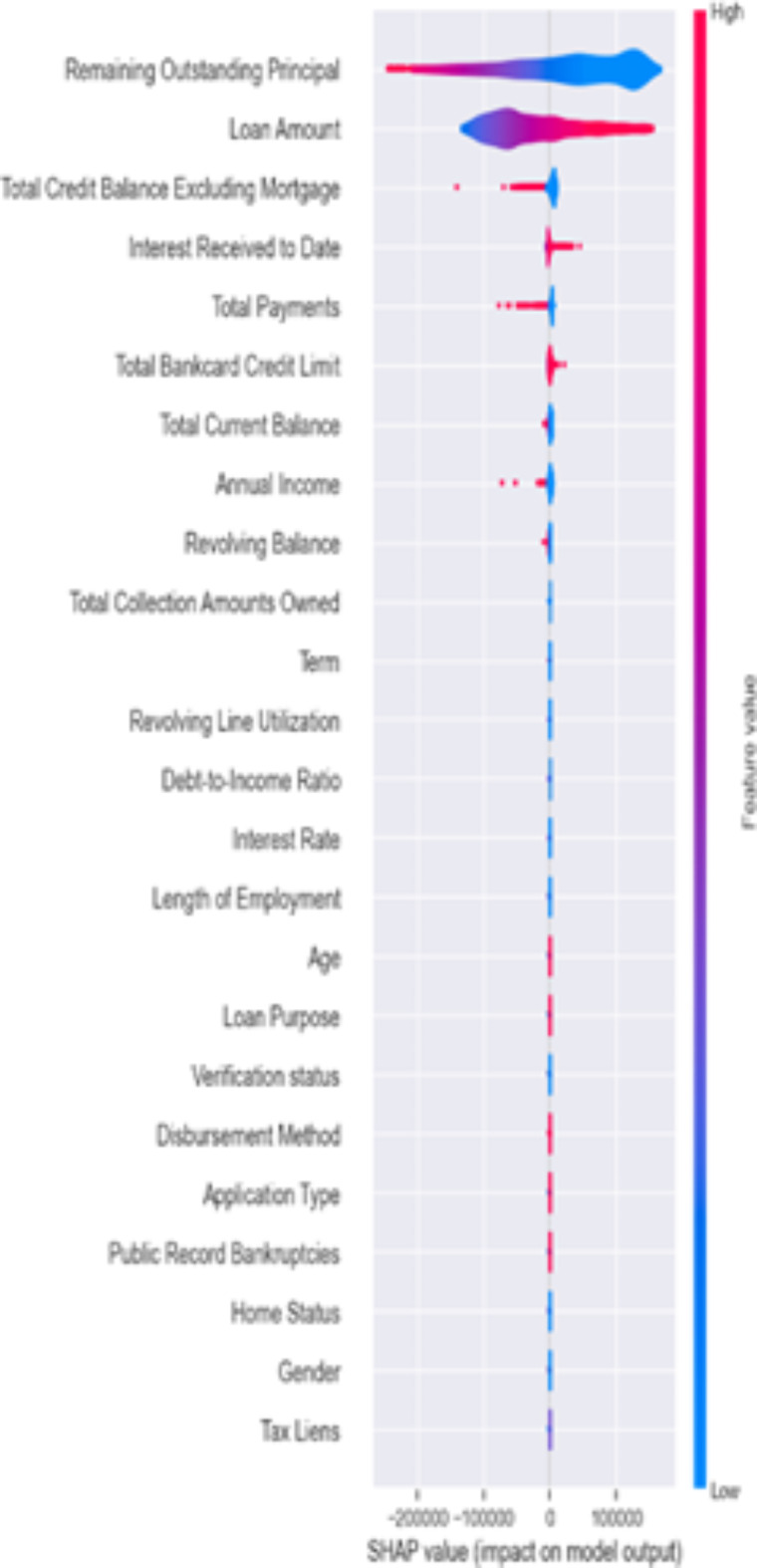
Features ranking Logistic regression model.

**Fig. 8:**
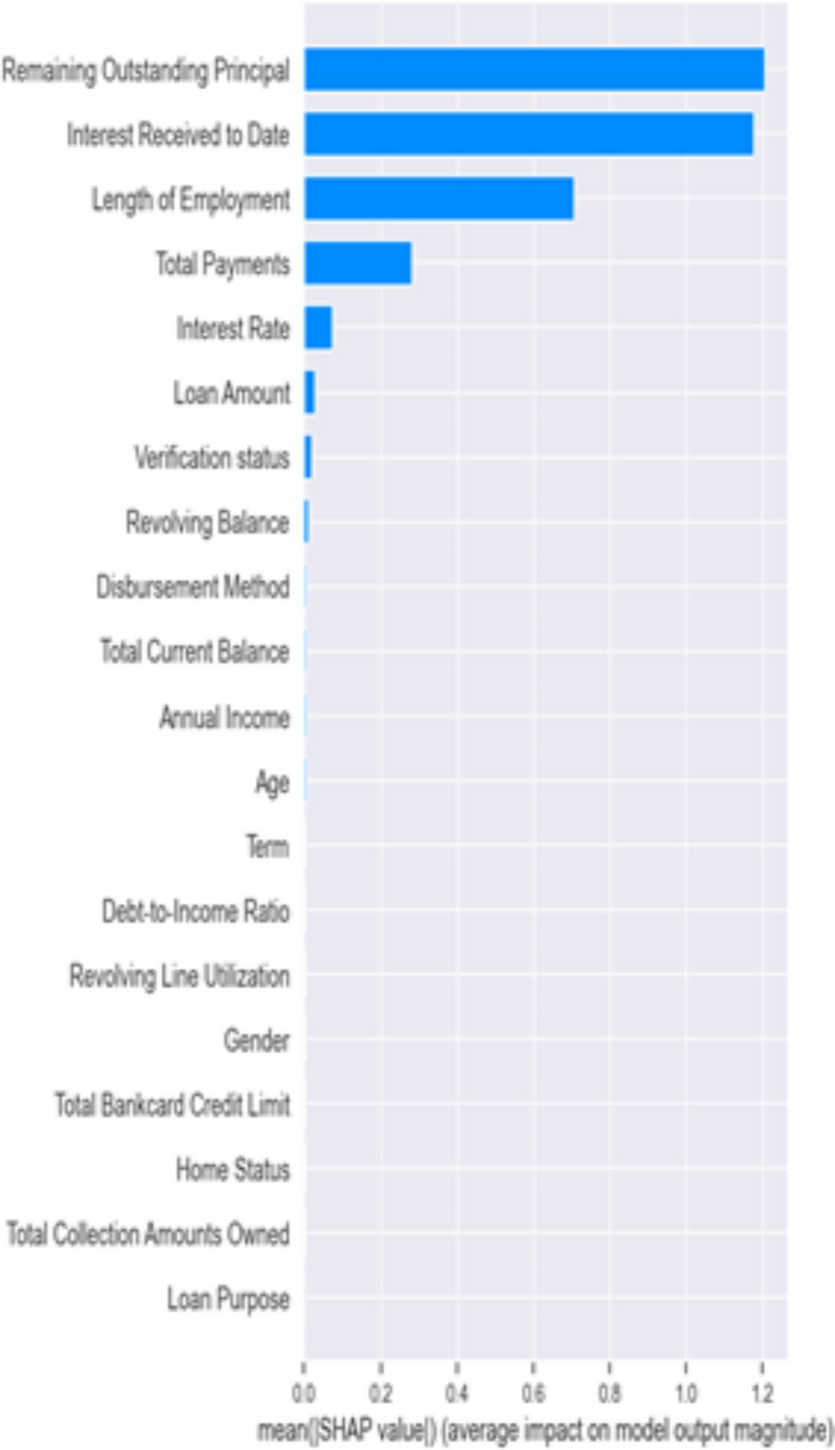
Features ranking $\:XGBoost$ model

Figures 6, 7 and 8 below presents the Global explainablity for the models. Global explainablity in machine learning-based credit scoring models is critically important because of the high-stakes nature of financial decision-making and the increasing regulatory scrutiny on automated decision systems. All features are shown in the order of global feature importance, the first feature is the most important, and the last is the least important feature. Figures 6, 7 and 8 reveal a significant discrepancy in how these models rank the importance of various features. For example, the $\:1DCNN$ and $\:XGBoost$ places the $\:interest\:rate$ as the fifth most critical feature, whereas the logistic regression (LR) model ranks it much lower at the fourteenth position. This variation in feature importance ranking, especially concerning the $\:interest\:rate$, highlights a fundamental difference in how these models perceive its influence on default prediction. How models prioritise certain features over others can affect decision-making, especially in tailoring financial products and managing risk. A model that significantly underestimates or overestimates the importance of features like interest rates may lead to suboptimal lending decisions, affecting both the lender’s risk exposure and the borrower’s financial well-being. While the $\:IDCNN$ and $\:XGBoost$ models appear to rank features similarly, $\:XGBoost$ classified half of the features as dummy players—i.e., their inclusion or exclusion does not change the model’s predictions. In these instances, their Shapley values are low or zero, indicating little to no importance.

**Fig. 9:**
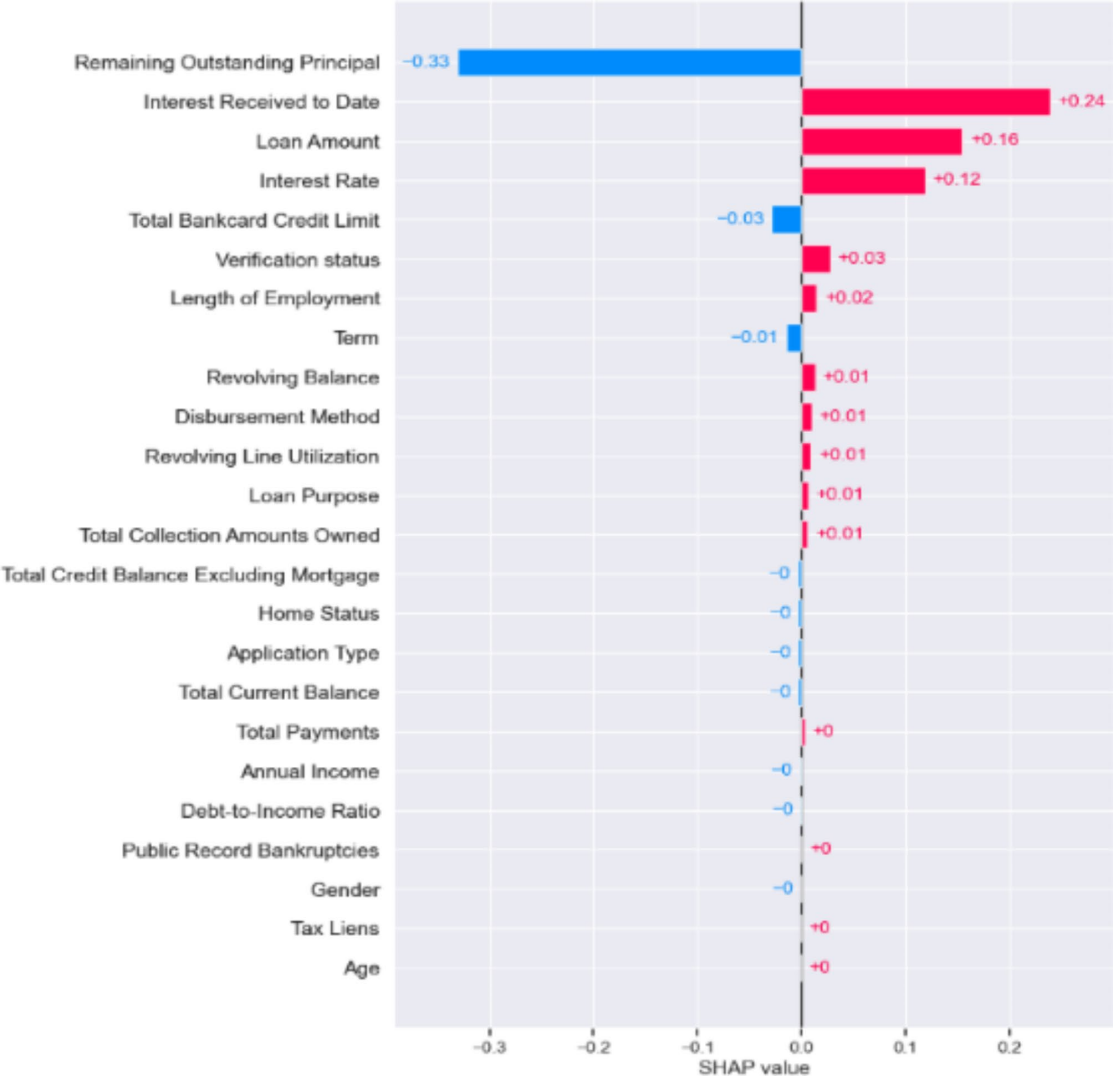
1DCNN local explainablity (customer 1).

**Fig. 10:**
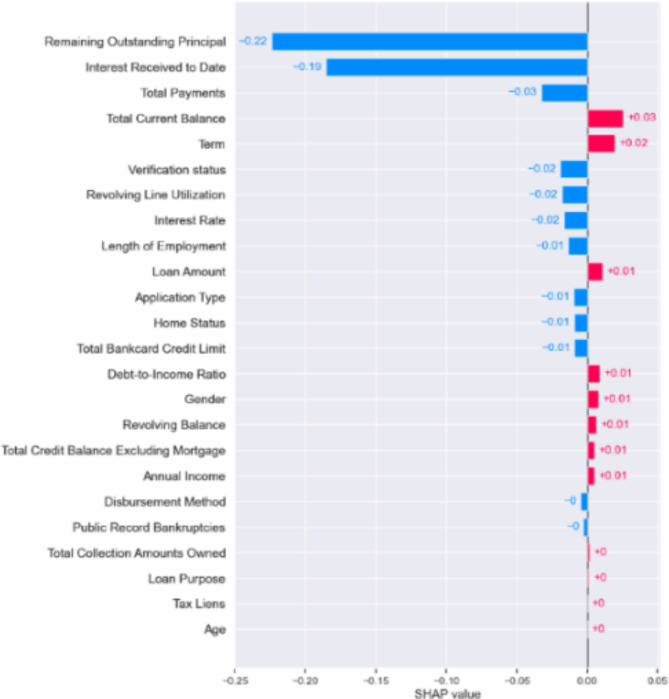
1DCNN local explainablity (customer 2).

In addition, we evaluated how much each feature has contributed to an individual targeted prediction (local explainablity). In other words, a credit risk analyst who wishes to understand the contribution of each input feature on the output of the model for a single observation (customer) can use the SHAP framework to achieve this. Figures 9 and 10 below shows the Shapley plot for two customers. A negative Shapley value for a feature regarding a specific prediction means that the feature contributes to shifting the prediction away from the “default” class towards the “no default” class, compared to the average prediction of the model. The bar length shows the SHAP value for each feature for the customers (observation). The blue bar shows a negative effect, while the red bar shows a positive effect. The absolute SHAP value reveals the feature with the most significant effect in predicting weather this customer will default or not. The relevance of local explainablity becomes evident when we compare the predictions for two customers in Figs. 9 and 10. For both customers, the model identified “*remaining outstanding principal*” as a crucial factor contributing to a lower probability of default. This indicates that as the remaining principal decreases, the likelihood of the customer defaulting on their loan diminishes, according to the model’s learned patterns. This finding aligns with financial logic—customers with less debt remaining might be less likely to default, possibly due to a shorter remaining loan term or their proven ability to make consistent payments. However, the model’s interpretation of “*interest received to date*” shows differences between the two customers. For customer 2, the amount of interest received so far is associated with a lower default probability than the model’s average prediction. This could indicate that customer 2 has been consistent in their payments, resulting in a substantial interest payment, which the model interprets as a positive indicator of financial stability and reduces the predicted risk of default.

On the other hand, for customer 1, the same feature—“*interest received to date*”—increases the probability of default, contrary to the effect observed for customer 2. This suggests that, for customer 1, the accrued interest might signal a different underlying issue, such as the customer having paid interest without significantly reducing the principal, possibly indicating financial strain or reliance on minimal payments. Alternatively, this could reflect a different loan structure or payment history the model has learned to associate with higher risk. This contrast in how the same feature affects predictions for different customers highlights the importance of local explainablity. It allows us to understand the specific context in which the model operates for each individual, providing insights that are not apparent from global model behaviour alone. For lenders, this means they can make more informed decisions, offering tailored advice or interventions based on the unique financial situation of each customer. Local explainablity ensures transparency for customers, helping them understand why certain decisions were made, which can build trust in the lending platform and its assessment process.

Achieving local explainablity is central in reducing bias in machine learning models^32^ because it allows us to see the variables driving the model’s decision. It plays a crucial role in reducing bias in lending, particularly regarding potentially discriminatory features like age and gender. Ensuring fairness in machine learning models, especially those used in sensitive applications such as credit risk assessment, is important. Biases in these models can lead to unfair outcomes, disproportionately affecting certain demographic groups and perpetuating systemic inequalities. The SHAP values in our model showed that for customer 1, both age and gender had minimal impact on the decision, with SHAP values of -0 and + 0, respectively. For customer 2, age had a similarly low SHAP value of + 0, and gender was slightly higher at + 0.01 but still ranked very low in significance, placing tenth from the bottom in terms of influence. The low SHAP values for age and gender indicate that the model does not heavily rely on these features when predicting the likelihood of default for these customers. This is a positive indicator of fairness in the model, suggesting that discriminatory factors do not disproportionately influence it. Furthermore, by providing these granular details, local explainablity enables model developers and auditors to take corrective actions if bias is detected. For instance, if age or gender were found to have a disproportionately high impact on predictions for some customers, it would signal the need for further investigation and potential re-training of the model, removing these two features to ensure that it does not unfairly penalise certain groups.

In our final analysis, we examined whether removing discriminatory features, specifically gender and age, from the hybrid model would affect its ability to classify outcomes accurately. This line of enquiry is essential in light of the U.S. Equal Credit Opportunity Act (ECOA). The ECOA is designed to regulate all entities that participate in credit decisions in their ordinary course of business, such as banks, retailers, credit unions and credit card issuers^33^. The Act, a Federal statute (15 U.S.C. § 1691), prohibits creditors from discriminating in any aspect of a credit transaction based on an applicant’s race, colour, religion, national origin, sex, marital status or age (if the applicant is legally capable of entering a contract). An equivalent Act in the U.K., is the Equality Act 2010, which replaced the previous discrimination legislation in Great Britain. The Act prohibits discrimination relating to sex, race, sexual orientation, religion or belief and age in the public and wider society^34^. The findings presented in Table 5 below, indicate that the hybrid model’s classification accuracy remains essentially unchanged (0.96) without these two variables. This suggests that gender and age do not play a significant role in the model’s predictions-see Figs. 6, 7, 8, 9 and 10, akin to “dummy players” that do not contribute to the outcome. Removing features like gender and age helps ensure that the model’s assessments are based on relevant financial behaviours rather than characteristics that could lead to unfair discrimination. This approach aligns with ethical guidelines and regulatory standards, promoting fairness and equality in credit decision making.

**Table 5:** Performance evaluation metrics hybrid model without gender and age.

Hybrid *1DCNNs-XGBoost*						
	Precision	Recall	F1-Score	$\:Accuracy$	$\:AUC$	$\:H-Score$
0	0.94	0.98	0.96	0.96	0.98	0.95
1	0.97	0.94	0.95			
$\:Macro\:Avg.$	0.96	0.96	0.96			
$\:Weighted\:Avg$	0.96	0.96	0.96			

**Fig. 11:**
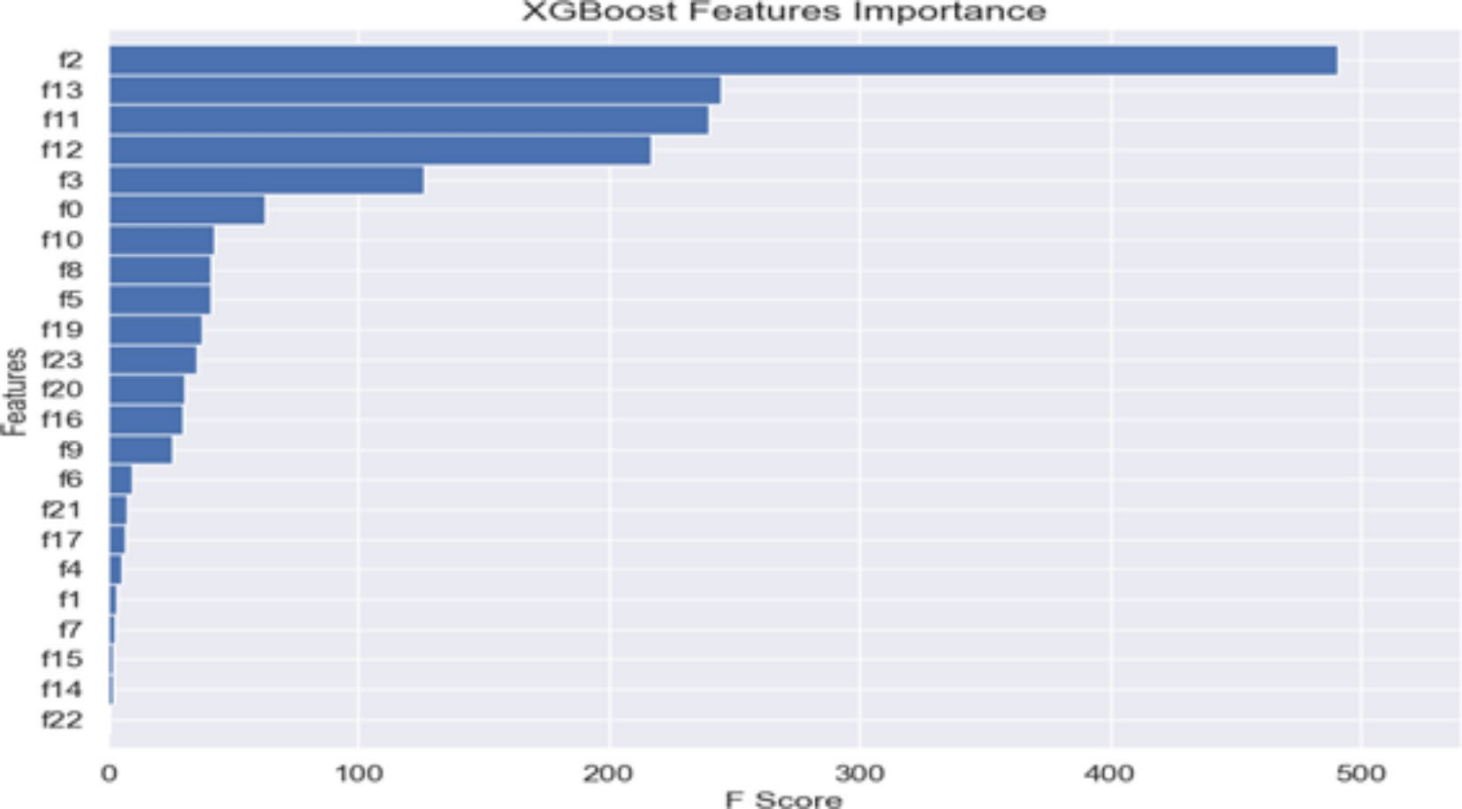
$\:XGBoost\:$features importance ranking. **Notes**: F0 denotes the Loan Amount, F1 Loan Term, F2 Interest Rate, F3 Length of Employment, F4 Home Status, F5 Annual Income, F6 Verification Status, F7 Loan Purpose, F8 Debt-to-Income Ratio, F9 Revolving Balance, F10 Revolving Line Utilization, F11 Remaining Outstanding Principal, F12 Total Payments, F13 Interest Received to Date, F14 Application Type, F15 Total Collection Amounts Owned, F16 Total Current Balance, F17 Public Record Bankruptcies, F18 Tax Liens, F19 Total Credit Balance Excluding Mortgage, F20 Total Bankcard Credit Limit, F21 Disbursement Method, F22 Gender, F23 Age.

Again, to test the robustness of the Shapely algorithm in ranking features importance, we used $\:XGBoost{\prime\:}s$ built-in feature importance functionality. We aimed to assess the contributions of age and gender in the classification of default and no default in our dataset further. Figure 11 shows that the built-in features ranking supports our earlier findings that age and gender do not contribute significantly to the classification outcomes. Specifically, gender, F22, had zero contributions, while age, F23, did not make it to the first ten features as in the IDCNN model.

While $\:XGBoost{\prime\:}s$ built-in features importance provides a quick way to gauge the relative importance of features, it is crucial to remember that this method is biased towards numerical features and features with a larger number of categories^35^. Since it measures importance based only on the specific structure of trees in $\:XGBoost$, its insights might not fully capture feature interactions or dependencies that model-agnostic methods like SHAP could reveal. In addition, the feature importance metrics provided by $\:XGBoost,$ such as weight, gain, and cover, are global measures. That is, these metrics are aggregated across all trees within the model and provide only overall features importance without showing how features affect individual predictions. SHAP, on the other hand assigns each feature an importance value for a particular prediction allowing us to understand how each feature in the input contributes to the final decision made by the model.

## Conclusion

This paper uses a novel architecture to address the “black box” nature of the ML-powered credit scoring model. Despite their predictive ability, they lack interpretability, which could hinder trust and regulatory compliance, especially in the financially sensitive area of credit risk analysis. To achieve our aim, we compared the classification accuracies and features extraction capabilities of a one-dimensional convolutional neural network $\:\left(1DCNN\right)$, a novel hybrid model, $\:XGBoost$ and logistic regression, on a peer-to-peer (P2P) consumer credit dataset. Our analysis demonstrated that the hybrid model outperformed the conventional models regarding classification accuracy. We achieved a classification accuracy of **96%** for the hybrid model, surpassing the $\:1DCNN{\prime\:}s$**94%**, $\:XGBoost$**94%** and logistic regression’s $\:92\mathbf{\%}$. Additionally, using the Shapley framework enhanced the transparency and interpretability of our model, enabling us to pinpoint and mitigate likely biases effectively. The implications of our work extend beyond academic discourse, offering tangible benefits for the credit industry by providing credit analysts with a tool for assessing loan default risks, positively leading to better-informed lending decisions. The hybrid model’s ability to handle features extraction and complex interactions makes it reliable in data-intensive financial environments. Our findings that removing age and gender from the hybrid model has a non-significant impact on predictive accuracy suggest that achieving fairness without compromising the model’s performance is possible. This is an encouraging result, indicating that models can be designed to reduce reliance on discriminatory features while still maintaining high predictive accuracy. However, a limitation of this study is that it primarily focuses on a specific dataset and context. Future research could explore applying this hybrid model to other lending scenarios, such as corporate or consumer lending, using diverse datasets. Additionally, future studies might investigate integrating various machine-learning techniques to identify effective strategies for balancing classification accuracy with interpretability and fairness. This broader exploration could further enhance our understanding of how to build fair and accurate models across different financial contexts.

## Appendix

**Table 6:** Variables definitions and measurements.

	Definition	Measurement
**Dependent Variable**		
$\:Loan\:status$	charged-offs, default, and late (31–120 days)	(1, if loan is in default and 0, if performing)
**Borrower Characteristics**		
$\:Emp\_Length$	Length of current employment	Years
$\:Annual\_Inc$	Annual Income	Thousand
$\:DTI$	Debt-to-Income ratio	Ratio
$\:Home\_owns$	Home ownership status	Rent = 1, Own outright = 2, Own with Mortgage = 3, Other = 4
$\:Pub\_Rec$	Public record bankruptcies	Categorical variable ….
$\:Disbursement\:method$	The method by which the borrower receives their loan.	Categorical variable
$\:Tax\:liens$	legal claim against the assets of a person who fails to pay taxes.	Thousands
$\:Age$	Measures the age of the loan customers	Years
$\:Gender$	Measures the gender of the loan customers	Binary (Meal or Female)
**Loan Characteristics**		
$\:Loan\_amt$	Loan Amount	Thousands (numeric)
$\:Term$	Loan Contract Term	Months
$\:Interest$	Interest rate on the loan	%
$\:Account\:balance$	Cash balance in borrow loan proposer’s account	Thousands (numeric)
$\:Verification\:status$	Source of income verified	Dummy (1 if income was verified, 0 = otherwise
$\:Application\:type$	Type of loan application	Individual or joint application
$\:Credit\:limit$	Credit limit on am account	Thousands (numeric)
$\:Loan\:purpose$	A category provided by the borrower for the loan request	Categorical variable
**Repayment Characteristics**		
$\:Revolving\:line\:utilization$	Amount of loan the borrower is using	Thousands (numeric)
$\:Total\:credit\:balance\:excluding\:mortgage$	Total credit balance without mortgage	Thousands (numeric)
$\:Revolving\:balance$	Total revolving credit	Thousands (numeric)
$\:Interest\:received\:to\:date$	Total interest payments received to date	Thousands (numeric)
$\:Total\:payments$	Total payments received to date for the funded amount	Thousands (numeric)
$\:Remaining\:outstanding\:principal$	Measures the loan principal still outstanding	Thousands (numeric)
$\:Principal\:received\:to\:date$	Measures the principal the firm has received to date	Thousands (numeric)
$\:Total\:current\:balance\:of\:all\:account$	The current balance of customer $\:i\:$in all accounts	Thousands (numeric)
$\:Average\:current\:balance\:all\:account$	The average balance of customer $\:i$ in all account	Thousands (numeric)
$\:Total\:open\:to\:buy\:on\:revolving\:bankcards$	Total amount that investors can buy on revolving bankcards	Thousands (numeric)

## Electronic supplementary material

Below is the link to the electronic supplementary material.

Supplementary Material 1

## Data Availability

No datasets were generated or analysed during the current study.

## Footnotes

1 In this study will have used interpretability and explainablity interchangeably.
