# Supplementary material for: Enhancing transparency and fairness in automated credit decisions: an explainable novel hybrid machine learning approach
Source: Sci Rep. 2024 Oct 24;14:25174. doi: 10.1038/s41598-024-75026-8 (PMC11502870; doi:10.1038/s41598-024-75026-8)
Supplement: Supplementary file 1 — Supplementary Material 1 [file 41598_2024_75026_MOESM1_ESM.docx]

**Appendix**.: Table 2: Definition and measurement of features

|  | Definition | Measurement |
| --- | --- | --- |
| **Dependent Variable** | |  |
| $Loan status$ | charged-offs, default, and late (31-120 days) | (1, if loan is in default and 0, if performing) |
| **Borrower Characteristics** | |  |
| $Emp\_Length$ | Length of current employment | Years |
| $Annual\_Inc$ | Annual Income | Thousand |
| $DTI$ | Debt-to-Income ratio | Ratio |
| $Home\_owns$ | Home ownership status | Rent=1, Own outright =2, Own with Mortgage =3, Other =4 |
| $Pub\_Rec$ | Public record bankruptcies | Categorical variable …. |
| $Disbursement method$ | The method by which the borrower receives their loan. | Categorical variable |
| $Tax liens$ | legal claim against the assets of a person who fails to pay taxes. | Thousands |
| $Age$ | Measures the age of the loan customers | Years |
| $Gender$ | Measures the gender of the loan customers | Binary (Meal or Female) |
| **Loan Characteristics** | |  |
| $Loan\_amt$ | Loan Amount | Thousands (numeric) |
| $Term$ | Loan Contract Term | Months |
| $Interest$ | Interest rate on the loan | % |
| $Account balance$ | Cash balance in borrow loan proposer’s account | Thousands (numeric) |
| $Verification status$ | Source of income verified | Dummy (1 if income was verified, 0 = otherwise |
| $Application type$ | Type of loan application | Individual or joint application |
| $Credit limit$ | Credit limit on am account | Thousands (numeric) |
| $Loan purpose$ | A category provided by the borrower for the loan request | Categorical variable |
| **Repayment Characteristics** | |  |
| $Revolving line utilization$ | Amount of loan the borrower is using | Thousands (numeric) |
| $Total credit balance excluding mortgage$ | Total credit balance without mortgage | Thousands (numeric) |
| $Revolving balance$ | Total revolving credit | Thousands (numeric) |
| $Interest received to date$ | Total interest payments received to date | Thousands (numeric) |
| $Total payments$ | Total payments received to date for the funded amount | Thousands (numeric) |
| $Remaining outstanding principal$ | Measures the loan principal still outstanding | Thousands (numeric) |
| $Principal received to date$ | Measures the principal the firm has received to date | Thousands (numeric) |
| $Total current balance of all account$ | The current balance of customer $i$in all accounts | Thousands (numeric) |
| $Average current balance all account$ | The average balance of customer $i$ in all account | Thousands (numeric) |
| $Total open to buy on revolving bankcards$ | Total amount that investors can buy on revolving bankcards | Thousands (numeric) |
